# Executive Function and Resilience as Mediators of Adolescents’ Perceived Stressful Life Events and School Adjustment

**DOI:** 10.3389/fpsyg.2019.00446

**Published:** 2019-02-28

**Authors:** Yuqing Zhang, Xing Zhang, Liwei Zhang, Cheng Guo

**Affiliations:** ^1^The Lab of Mental Health and Social Adaptation, Faulty of Psychology, Southwest University, Chongqing, China; ^2^Research Center of Mental Health Education, Southwest University, Chongqing, China; ^3^Faulty of Psychology, Southwest University, Chongqing, China; ^4^Physical Education Department, Luoyang Vocational & Technical College, Luoyang, China

**Keywords:** stressful life events, school adjustment, executive function, resilience, adolescents

## Abstract

This study investigated psychological mechanisms underlying the relationship between stressful life events and school adjustment in Chinese adolescents. The Adolescent Self-rating Life Events Checklist, the Adolescent Executive Function Scale, the Chinese version of the Resilience Scale, and the School-adjustment Scale were administered to 1101 Chinese adolescents (465 males, 636 females), aged 11–19 years, from three secondary schools. Results from serial mediation analysis revealed that perceived stressful life events could affect school adjustment through the mediation of executive function and resilience. The mediation effect contained three paths, the separate mediation effect of executive function, the separate mediation effect of resilience, and the serial mediation effect of executive function and resilience. These findings provide valuable insights into the effect of perceived stressful life events on school adjustment of Chinese adolescents, and suggest that, the researchers and educators could enhance school adjustment in vulnerable groups by improving their executive function and resilience.

## Introduction

We will encounter various stressful events in our daily life. Indeed, stressors begin to affect individuals from birth (Kramer et al., 2009), and occur in the daily life of virtually every individual (Compas et al., 1993). Stressful life events refer to life experiences that result in changes in an individual’s life and those that necessitate coping and adjustment strategies (Compas, 1987). Adolescence is an important transition in academic, cognitive, social, physiological, and physical change (Arnett, 1999), and is a fragile developmental stage characterized by exposure to stressful life events and their debilitating mental health effects (Byrne et al., 2007). The stressful life events among adolescents have attracted the attention of many researchers (Dupéré et al., 2018; Han et al., 2018; Humphreys et al., 2018). Furthermore, a large amount of studies showed that stressful life events can exert profound influence on an individual’s maladjustment (Sandler et al., 1994; Rod et al., 2009), and stressful life events in adolescence are correlated with many negative outcomes, such as decreased well-being, impaired mental health, anxiety and depression (Troy and Mauss, 2011). Other studies further pointed that the stressful life events played a role in triggering high school dropout (Dupéré et al., 2018), and higher levels of perceived stress were associated with decreased hippocampal volume in adolescent (Piccolo and Noble, 2017). Thus, the stressful life events have negative effects on the physical and mental health of adolescents. Adolescence period is in school-age, and the adolescents spend most of their time in school activities. Thus, school adjustment of adolescents is an important problem that the whole society need pay special attention to (Maclean et al., 2016; Chen et al., 2019). However, the mediating processes that account for the association between perceived stressful life events and school adjustment are still being identified. Therefore, it is urgent to explore the association between perceived stressful life events and school adjustment and the psychological mechanisms behind this association among adolescence.

### Perceived Stressful Life Events, Executive Function, and School Adjustment

Executive function refers to a family of top-down mental processes that are essential for tasks requiring concentration and attention, performed in such instances when relying on instinct or intuition would be ill advised or insufficient. Core executive functions include inhibition control, working memory, and cognitive flexibility (Diamond, 2013). Prior studies provide indirect support for the prediction that stressful life events impair executive function in adolescence. For example, many studies showed that early life stress exposure was associated with poorer adult cognitive function in memory domains and executive function in healthy and psychopathology populations (Navalta et al., 2006), and individuals who reporting predictive early life stress exhibited poorer processing speed and working memory performance (Saleh et al., 2017). In addition, Pukay-Martin et al. (2003) found that, for HIV-positive subjects, stressful life events were related to poor performance on measures of executive function, attention, and processing speed, while positive life events were related to better performance. In addition to behavioral evidence, evidence from neuroimaging study also suggested that, greater activation in brain regions responsible for executive functions accounted for the association between exposure to chronic stress and less use of adaptive coping among adolescents (Reising et al., 2018). It’s not hard to come to conclusion that, stressful life events may have a negative effect on executive function, the more stressful life events the individuals perceived, the individuals may show worse executive function performance. In general, impaired executive function is viewed as maladjusted cognitive activity and has been shown to be related to anxiety and depression (Brosschot et al., 2006; Greenaway and Howlin, 2010). In addition, other studies pointed to the effects of executive function on children’s academic performance, specifically in reading, mathematics, and interpersonal relationships (Clark et al., 2002; Blair and Razza, 2007; Fitzpatrick et al., 2014), as well as late adolescents’ academic performance (Baars et al., 2015). More importantly, the prevailing argument is that impairments in executive function can lead to significant and lasting adjustment difficulties (Biederman et al., 2004; Ellis et al., 2004). Consistent with this argument, several longitudinal studies suggested that executive function was a predictor of children’s school adjustment (Jacobson et al., 2011; Masten and Tellegen, 2012) and was regard as a determinant of academic performance in late adolescents university students (Baars et al., 2015). Accordingly, perceived more stressful life events may lead to impairments in execution function, which in turn may affect the school adjustment of the adolescents. However, most of studies in the domain of executive function were conducted by experimental paradigm. Laboratory studies on executive function have been developed comprehensively, but executive function are less measured by self-reported questionnaires (Gioia et al., 2000; Baars et al., 2015). Furthermore, because of its practicality and flexibility, the self-reported executive function scale can get more observable and self-insight data than laboratory tests (Barkley and Murphy, 2011; Baars et al., 2015). Self-reported executive function scale provides practitioners a reliable and effective assessment tool to assess the level of executive function. Accordingly, executive function, being measured by questionnaires and being considered as a fundamental human cognitive factor, might play a significant role in the influence between perceived stressful life events and school adjustment among adolescents.

### Perceived Stressful Life Events, Resilience and School Adjustment

Resilience can be broadly defined as the potential or manifested capacity of a dynamic system to adjust successfully to disturbances that threaten the function, survival, or development of the system (Masten, 2015). As Masten (2015) has suggested, the research in the domain of resilience mainly focuses on adjustment under challenging circumstances. Although everyone will encounter various life stresses, not everyone will show negative outcomes in the face of stress, and some exhibit resilience even in the face of high levels of stress exposure. Many studies found the negative association between stressful life events and resilience. For example, Cole et al. (2015) found a negative relationship between stress of academic performance and ego resilience, which provides preliminary evidence for this relationship; other study showed that the resilience level of Sichuan earthquake survivors was lower than that of those who didn’t experience the earthquake (Ni et al., 2015). More importantly, many recent studies from adolescent population also indicated that, the stressful life events were negatively associated with resilience (Anyan and Hjemdal, 2016). Thus, stress may have an adverse impact on an individual’s resilience.

In addition, a large scale of studies showed that, resilience was positively relate to people’s mental health (Catalano et al., 2011; Li et al., 2012) and quality of life (Migerode et al., 2012), while was negatively associated with depression or anxiety (Anyan and Hjemdal, 2016). As an important stress coping resource, resilience also contributes to psychological and behavioral adjustment. For example, Luthar et al. (2000) found that, resilience was particularly important in forming psychosocial adjustment (e.g., at-risk children who appeared resilient, based on high academic grades, also showed positive adjustment based on persevering classroom behaviors as perceived by others). Other studies further found that, resilience had a predictive role in school adjustment among elementary school students (Lee et al., 2014) and middle school students (Zhu et al., 2012). More importantly, a recent study found resilience played a mediator role of the association between academic stress and school life adjustment among adolescent (Kim et al., 2018), which provides more direct evidence for the possible mediating role of resilience on the association between perceived stressful life events and school adjustment in adolescents. Thus, stressful life events affect resilience, and resilience has an influence on school adjustment, it is reasonable to predict that, resilience plays a mediator in the relationship between perceived stressful life events and school adjustment in adolescents.

### Perceived Stressful Life Events, Executive Function, Resilience and School Adjustment

Although perceived stress has a detrimental effect on resilience, Masten and Tellegen (2012) argued that, executive function provided much of the capacity for resilience in humans. Benight and Cieslak (2011) argued that, the cognitive determinants played important role in successful adjustment, and proposed a self-efficacy based model of resilience. According to the self-efficacy based model of resilience, the focus on cognitive factors could easily apply in theoretical models to enhance our understanding of resilience. Due to executive function’s effect on goal-oriented behavior, having effective executive function may enhance individuals’ sense of self-efficacy, or the feeling that one has abilities to accomplish tasks. For example, executive function had been found to be related to self-efficacy beliefs regarding exercise (McAuley et al., 2011) in older adults, and driving ability (Rike et al., 2015) in individuals with brain injury. As a part of broader motivational system, the self-efficacy may be a key component of resilience (Masten, 2014). These studies provide preliminary evidence for the relationship between executive function and resilience from theoretical and empirical aspects. Additionally, Greenberg (2006) suggested that the interventions aimed at improving children’s executive function could promote resilience, implying the important role of executive function ability in resilience. More importantly, executive function was found to be associated with resilience in adolescents with depressed mothers (Davidovich et al., 2016), as well as in first-year undergraduate students (McKee, 2017). Thus, the concrete theoretical and empirical bases support the close association between executive function and resilience. Further, as discussed previously, stressful life events may lead to impaired executive function and resilience may contribute to better school adjustment in adolescence. Therefore, it is reasonable to predict that, the less stressful life events the adolescents perceived may be associated with better executive function, executive function has positive effect on adolescents’ resilience, and resilience may contribute to better school adjustment in adolescence.

### The Current Study

In sum, the purpose of this study is to examine the influence of stressful life events on the school adjustment of adolescents and the potential mediators (e.g., execution function and resilience) behind this association. Adolescence is a fragile developmental stage characterized by exposure to stressful life events and the adolescents’ school adjustment is an important problem that the whole society need pay special attention to (Lan et al., 2019), exploring the potential psychology mechanisms behind the association between stressful life events and school adjustment in adolescence may contribute to reduce the negative impact of stressful life events on their school adjustment, thus having important theoretical and practical significance (Benight and Cieslak, 2011). Based on the literature review, we hypothesized that:

H1: Executive function would mediate the impact of stressful life events on school adjustment.
H2: Resilience would mediate the impact of stressful life events on school adjustment.
H3: Executive function and resilience would serially mediate the impact of stressful life events and school adjustment. This mediation model contains three paths.

## Materials and Methods

### Participants

Participants were 1175 adolescents randomly recruited from three secondary schools in the southwest of China. Because the invalid questionnaires of some participants, a total of 74 adolescents were excluded from analyses. Thus, data from 1101 adolescents were used after data screening procedures (465 males, 636 females, aged 11–19 years, *M* = 14.77). All of adolescents and their parents had provided written informed consent before data collection, and then all of adolescents filled out all the paper questionnaires in pen. This study was carried out in accordance with the recommendations of the Human Ethics Committee of the Southwest University. All the adolescents gave written informed consent in accordance with the Declaration of Helsinki. The protocol was approved by the Human Ethics Committee of the Southwest University.

### Measures

#### Perceived Stress

The Adolescent Self-rating Life Events Checklist (ASLEC; Xin and Yao, 2015) was originally used to assess the severity of recent negative life events during the past 6 months. The respondents rated the impact of each negative life event on a 5-point Likert scale (1 = *not at all* to 5 = *extremely severe*). This measure incorporates 26 items, including five dimensions: being punished, loss, relationship pressure, learning pressure, and adaptation problems (Xin and Yao, 2015). The higher the score, the more stressful life events were perceived. This scale has been found to be reliable and valid among Chinese adolescents (Xin et al., 2016). In current study, the Cronbach’s alpha was 0.934, the composite reliability was 0.896, the average variance extracted was 0.633 and the MacDonald’s omega was 0.855, 95% CI = 0.837 to 0.869.

#### Executive Function

The Adolescent Executive Function Scale (Huang et al., 2014) was originally used to assess executive function status in adolescents. This scale contains 21 items that consist of three factors, namely, inhibition control, cognitive flexibility, and working memory. The respondents rated how often the specific behaviors happen in the past 6 months on a 3-point Likert scale (1 = *not at all* to 3 = *often*). Higher scores on this metric typically indicate worse executive function performance. However, in the current study, all items were reverse-scored so that high scores indicated better executive function performance. The Cronbach’s alpha in the current study was 0.852, the composite reliability was 0.793, the average variance extracted was 0.561, and the MacDonald’s omega was 0.850, 95% CI = 0.832 to 0.864.

#### Resilience

Resilience was originally assessed with the Chinese version of the Resilience Scale (RS; Wagnild and Young, 1993), which has been widely used in China and has been shown to be a reliable and valid measure in Chinese adolescents (Zheng et al., 2011). This scale consists of 25 items arranged in two subscales: personal competence and acceptance of the self and life. The higher the score, the higher the resilience level of the respondents. Participants rated the extent to which each item reflected them on a 7 - point Likert scale (1 = *disagree* to 7 = *agree*). In this study, the Cronbach’s alpha reliability coefficient was 0.880, the composite reliability was 0.908, the average variance extracted was 0.831, and the MacDonald’s omega was 0.855, 95% CI = 0.841 to 0.869.

#### School Adjustment

School adjustment was originally assessed with the School-adjustment Scale (Sung et al., 2014) and contains 21 statements. The questionnaire includes three dimensions: teacher-student relationships, peer relationships, and self-adjustment. A 6-point Likert scale (1 = *strongly disagree* to 6 *strongly agree*) was used to indicate the agreement level of each statement with regard to school adjustment. The higher the score, the higher the school adjustment level of the respondents. In the current study, the Cronbach’s alpha value is 0.852, the composite reliability was 0.837, the average variance extracted was 0.631, and the MacDonald’s omega was 0.855, 95% CI = 0.838–0.869.

### Analytic Strategy

Our analytic approach involved two steps. Prior to the examination of the hypothesized mediational model regarding the mediating effects of execution function and resilience on the relation between perceived stressful life events and school adjustment, descriptive statistics and correlations were conducted with for all variables in the total sample by SPSS 21.0. Then, a two-step procedure was applied to analyze the mediation effect (Anderson and Gerbing, 1988; Zhang et al., 2016). Firstly, the measurement model, which involved four latent variables, was tested to assess the goodness of fit represented by its explicit indicators. Secondly, if the index of measurement model met the requirements, the maximum likelihood estimation examined the structural equation modeling (SEM). Mplus 7.4 was used to evaluate the hypothetical model’s data fit (Muthén and Muthén, 1998/2012). According to previous studies (Schafer and Graham, 2002; Graham, 2009; Curtis et al., 2016), missing data were handled using the full information maximum likelihood (FIML) procedure. SEM was performed using the robust maximum likelihood (MLR) estimator to account for identified data non-normality. Indirect effects were tested using bootstrapping procedures (Preacher and Hayes, 2008).

We used the following indices to evaluate the model’s data fit: the confirmatory fit index (CFI), the Tucker-Lewis index (TLI), the root mean square error of approximation (RMSEA), and the self-reunion multiple regression (SRMR). According to previous study, values >0.90 for the CFI and TLI, and <0.6 and <0.8 for the RMSEA and SRMR, respectively, were considered to indicate good model fit (Hu and Bentler, 1999).

## Results

### Descriptive Statistics and Correlations of Variables

Table 1 presents the descriptive statistics, including means, standard deviations, and Pearson’s bivariate correlations for all observed variables. Executive function was positively correlated with resilience, and resilience was positively correlated with school adjustment. Although other variables are statistically significantly interrelated (*p* < 0.01), their correlation coefficients were less than 0.30, which cannot be considered as a significative correlation.

**Table 1:** Descriptive statistics and intercorrelations between observed variables.

Variables	*M*	*SD*	1	2	3	4	5	6	7	8	9	10	11	12	13
1. Being punished factor	2.05	1.12	-												
2. Loss factor	2.22	1.17	0.56^∗∗^	-											
3. Relationship pressure factor	3.23	1.05	0.48^∗∗^	0.34^∗∗^	-										
4. Learning pressure factor	3.32	1.04	0.46^∗∗^	0.44^∗∗^	0.47^∗∗^	-									
5. Adaption pressure factor	2.41	0.96	0.51^∗∗^	0.53^∗∗^	0.51^∗∗^	0.51^∗∗^	-								
6. Inhibitory control	2.23	0.46	-0.15^∗∗^	-0.14^∗∗^	-0.31^∗∗^	-0.26^∗∗^	-0.22^∗∗^	-							
7. Cognitive flexibility	2.21	0.40	-0.14^∗∗^	-0.18^∗∗^	-0.27^∗∗^	-0.34^∗∗^	-0.31^∗∗^	0.33^∗∗^	-						
8. Working memory	2.23	0.51	-0.18^∗∗^	-0.21^∗∗^	-0.25^∗∗^	-0.23^∗∗^	-0.22^∗∗^	0.40^∗∗^	0.30^∗∗^	-					
9. Personal competence	4.58	0.84	-0.11^∗∗^	-0.08^∗∗^	-0.19^∗∗^	-0.17^∗∗^	-0.21^∗∗^	0.33^∗∗^	0.39^∗∗^	0.32^∗∗^	-				
10. Acceptance of self and life	4.58	0.84	-0.15^∗∗^	-0.11^∗∗^	-0.20^∗∗^	-0.20^∗∗^	-0.23^∗∗^	0.22^∗∗^	0.24^∗∗^	0.20^∗∗^	0.67^∗∗^	-			
11. Peer relationship	4.22	0.75	-0.11^∗∗^	-0.12^∗∗^	-0.25^∗∗^	-0.25^∗∗^	-0.21^∗∗^	0.38^∗∗^	0.34^∗∗^	0.29^∗∗^	0.50^∗∗^	0.41^∗∗^	-		
12. Teacher-student relationship	2.96	0.76	-0.10^∗∗^	-0.12^∗∗^	-0.19^∗∗^	-0.19^∗∗^	-0.33^∗∗^	0.28^∗∗^	0.28^∗∗^	0.18^∗∗^	0.39^∗∗^	0.32^∗∗^	0.40^∗∗^	-	
13. Self-adjustment	4.00	0.85	-0.10^∗∗^	-0.10^∗∗^	-0.28^∗∗^	-0.28^∗∗^	-0.30^∗∗^	0.31^∗∗^	0.39^∗∗^	0.24^∗∗^	0.57^∗∗^	0.53^∗∗^	0.54^∗∗^	0.46^∗∗^	-


*^∗∗^*p* < 0.01.*

### Measurement Model

Confirmatory factor analysis (CFA) was conducted to test the measurement model comprising the four interrelated latent variables: stressful life events, executive function, resilience, and school adjustment. The latent stressful life events variables were described as a being punished factor, loss factor, relationship pressure factor, learning pressure factor, and adaptation problem factor. The latent executive function variables were described by inhibitory control, working memory, and cognitive flexibility. The resilience latent variable was described by personal competence, and acceptance of self and life. The school adjustment variable was described by teacher-student relationships, peer relationships, and self-adjustment. The measurement model fit the observed data well: χ^2^= 420.324, df = 59, χ^2^/df = 7.124, CFI = 0.925, TLI = 0.901, SRMR = 0.051, and RMSEA = 0.075.

### Structural Model

**FIGURE 1:**
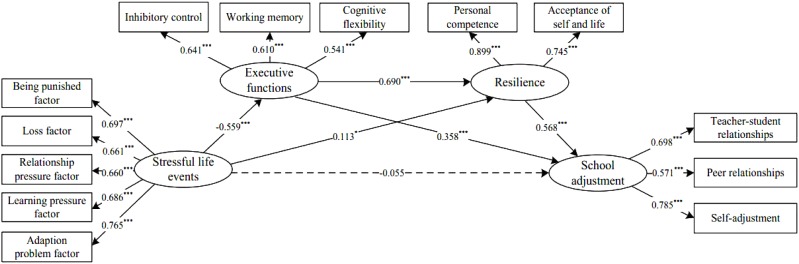
Standardized parameter estimates of the final model. ^∗∗∗^*p* < 0.001, ^∗^*p* < 0.05.

The model examined the associations between stressful life events, executive function, resilience, and school adjustment. Results showed acceptable data fit, χ^2^= 420.324, df = 59, χ^2^/df = 7.124, CFI = 0.925, TLI = 0.901, SRMR = 0.051, and RMSEA = 0.075. Specifically, the results provided support for H1 [i.e., executive function mediates the impact of stressful life events on school adjustment (β = -0.200, *SE* = 0.041, *p* < 0.001)], H2 [i.e., resilience mediates the impact of stressful life events on school adjustment (β = 0.065, *SE* = 0.029, *p* = 0.025)], and H3 [i.e., stressful life events exert a non-significant indirect effect on adolescent’s school adjustment through the three-path mediation effect of executive function and resilience (β = -0.055, *SE* = 0.042, *p* = 0.191)] (see Figure 1). We subsequently used Mplus’ model constraint command to create auxiliary variables and used bootstrapping in order to compare the mediation effects (Muthén and Muthén, 1998/2012; Preacher and Hayes, 2008). Executive function affected school adjustment more strongly than resilience (β = -0.265, *SE* = 0.054, *p* < 0.001, CI = -0.207 to -0.073; Table 2).

**Table 2:** Standardized indirect effects from stressful life events to school adjustment.

Indirect effect	β (standardized indirect effect)	*SE*	*p*	95% CI standardized indirect effect
From stressful life events to school adjustment via executive functions	-0.200	0.041	<0.001	-0.259, -0.103
via resilience	0.065	0.029	0.025	-0.013, -0.008
via executive functions and resilience	-0.219	0.030	<0.001	-0.231, -0.118


## Discussion

The purpose of present study was to explore the role of executive function and resilience in the relationship between perceived stressful life events and school adjustment among adolescents. Consistent with our hypotheses, perceived stressful life events affected school adjustment through the mediation of executive function and resilience. The mediation effect contained three paths: the separate mediation effect of executive function, the separate mediation effect of resilience, and the serial mediation effect of executive function and resilience. The serial mediation model indicated that, the experience of multiple stressful life events impeded the development of executive function, adversely affected the formation of resilience, and led to poor school adjustment among adolescents, which is consistent with results reported in prior studies (Best et al., 2009; Duckworth et al., 2012).

The findings from the current study revealed the mediating effect of executive function on the association between perceived stressful life events and school adjustment among adolescents. The result is consistent with previous studies regarding the negative link between stressful life events and executive function (Pukay-Martin et al., 2003; Saleh et al., 2017), the positive link between executive function and school adjustment (Jacobson et al., 2011; Masten and Tellegen, 2012; Fitzpatrick et al., 2014), and the close link between stressful life events and negative outcomes during adolescence (Nishikawa et al., 2018). A large scale of studies indicated that, executive function contributed to successful adjustment to school during childhood (e.g., school readiness and subsequent academic achievement) (Espy et al., 2004; Bull et al., 2008; Clark et al., 2010; Welsh et al., 2010; Hughes and Ensor, 2011; Neuenschwander et al., 2012), and academic success during early adolescence (Lafavor, 2018). More importantly, other study revealed the mediating role of executive function on the association between poverty and academic delays (Nesbitt et al., 2013). Obviously, poverty could be regarded as stressful life event. The current study confirms previous findings in children and adults are also applicable to the adolescent, and further show that perceived stressful life events have a negative influence on school adjustment of the adolescents via affecting executive function. Thus, the findings from the current study suggest that, the more stressful life events the adolescents perceived, may lead to worse executive function performance, which, in turn, have negative effects on the school adjustment.

The findings from the current study also indicate the mediating effect of resilience on the association between perceived stressful life events and school adjustment among adolescents. Some previous studies found that, the more stressful life events could lead to poorer resilience (Anyan and Hjemdal, 2016). Contrary to previous studies, the findings from current study show that, stressful life events could positively predict resilience among adolescents. Inconsistency in findings may be due to differences in measuring tools for variables and heterogeneity of participants with regard to age. In general, the stressful life events that individuals perceived in daily life belong to appropriate stress, and these appropriate stresses may help to develop adolescents’ resilience to stress. According to the challenge model of resilience, when appropriately exposed to risk that can be overcome, the individual, could learn to deploy resources to overcome future stress (Fergus and Zimmerman, 2004), which implies the special significance of appropriate stress on developing individuals’ resilience. The findings from present study seem to support the challenge model of resilience. Indeed, recent adversity may negatively affect individuals in the short term, while prior experience may yield a greater propensity for resilience over a prolonged period (Seery, 2011). In addition, previous research found a mediator role of resilience in the association between academic stress on school life adjustment among adolescent (Kim et al., 2018), and a moderating role of resilience on the relationship between perceived stress and binge eating symptoms among young adult women (Thurston et al., 2018). These studies demonstrate the important role of resilience on the relationship between stress and mental health. The current study confirms previous findings, and shows that, resilience is another intervening variable between perceived stress and school adjustment.

More interestingly, the current study suggests that, the perceived stressful life events may affect school adjustment of adolescents via the serial mediation effect of executive function and resilience, which emphasizes the serial effect of executive function and resilience on the association between stressful life events and school adjustment. The finding that executive function predicts resilience is consistent with previous research (Davidovich et al., 2016; McKee, 2017), and further supports the notion that the poor executive function may represent a risk factor that leads individuals to be less resilient to life stresses, which can have a negative impact on school adjustment. Additionally, previous work demonstrated that stressful life events could predict poorer executive function (Pukay-Martin et al., 2003), had influence on an individual’s maladjustment (Sandler et al., 1994; Rod et al., 2009), and were correlated with many negative outcomes (e.g., anxiety and depression) (Troy and Mauss, 2011). Furthermore, other study suggested that the association between stressful life events and teenage students’ social adjustment can be explained in part by social problem-solving skill (Yang et al., 2012), implying that stressful life events may have negative effect on cognitive ability. More importantly, many previous researches found the important effect of executive function on resilience (Davidovich et al., 2016; McKee, 2017), and the important role of resilience on the association between perceived stress and mental health (García-Izquierdo et al., 2018; Thurston et al., 2018). The present study extends previous works by demonstrating that, stressful life events are associated with poorer executive function, which in turn predicts resilience and school adjustment in adolescents. Overall, these findings increase our understanding of how stressful life events might be associated with school adjustment among adolescents.

The current study is not without limitations, which also implies suggestions for future research. First, since the study was cross-sectional, it precluded deriving any causal relationship between the variables. To address this issue and facilitate a better understanding of causal mechanisms, future research should conduct longitudinal, experimental or intervention studies. Second, the present study is one of the few studies to utilize a self-reported executive function scale among adolescents. In order to achieve a better understanding of the role of executive function in the relationship between perceived stressful life events and school adjustment among adolescents, future research should compare the differences between experimental and self-report methods in adolescent population. Third, in the present study, participants completed the questionnaires with pen and paper, questionnaire survey was conducted in a physical way may have some limitations, that is, lack of control of the time dedicated to each response and some data encoding errors, etc.,. Some online questionnaire survey App (e.g., SurveyMonkey) could overcome these limitations. In order to obtain more scientific and rigorous research data, online questionnaire survey App should be used more often in the future. Forth, the present study was conducted in the background of Chinese population, the findings cannot provide differences between Chinese population and the population of the West in the psychological mechanisms underlying the relationship between stressful life events and school adjustment. Therefore, cross-cultural studies should be conducted to compare the differences between eastern and western population in the future.

## Conclusion

In conclusion, to the best of our knowledge, the current study represents a new attempt to explore executive function and resilience as the underlying mechanisms in the relationship between perceived stressful life events and school adjustment among adolescents. Furthermore, as the significant indirect effects of perceived stressful life events on school adjustment separately via executive function or resilience, and a serial mediation effect of executive function and resilience were found, the current study also sheds light on how adolescents who perceived more stressful life events achieve worse school adjustment. The findings from the current study are significantly meaningful for conducting effective measures to break the cyclical relationship between stressful life events and school adjustment, thus having some practical implications.

## Author Contributions

YZ and CG collected the data together. YZ, XZ, and CG analyzed the results and wrote the manuscript. LZ participated in the revision of the manuscript.

## Conflict of Interest Statement

The authors declare that the research was conducted in the absence of any commercial or financial relationships that could be construed as a potential conflict of interest.

## References

[B1] Anderson J. C. Gerbing D. W. (1988). Structural equation modeling in practice: a review and recommended two-step approach. *Psychol. Bull.* 103 411–423. 10.1037/0033-2909.103.3.411

[B2] Anyan F. Hjemdal O. (2016). Adolescent stress and symptoms of anxiety and depression: resilience explains and differentiates the relationships. *J. Affect. Disord.* 203 213–220. 10.1016/j.jad.2016.05.031 27310100

[B3] Arnett J. J. (1999). Adolescent storm and stress, reconsidered. *Am. Psychol.* 54 317–326. 10.1037/0003-066X.54.5.317 10354802

[B4] Baars M. A. E. Marije N. B. Tonnaer G. H. Jelle J. (2015). Self-report measures of executive functioning are a determinant of academic performance in first-year students at a university of applied sciences. *Front. Psychol.* 6:1131. 10.3389/fpsyg.2015.01131 26300823 PMC4525669

[B5] Barkley R. A. Murphy K. R. (2011). The nature of executive function (EF) deficits in daily life activities in adults with ADHD and their relationship to performance on EF tests. *J. Psychopathol. Behav. Assess.* 33 137–158. 10.1007/s10862-011-9217-x

[B6] Benight C. C. Cieslak R. (2011). “Cognitive factors and resilience: How self-efficacy contributes to coping with adversities,” in *Resilience and Mental Health: Challenges Across the Lifespan*, eds Southwick S. M. Litz B. T. Charney D. (Cambridge: Cambridge University Press) 45–55. 10.1017/CBO9780511994791.005

[B7] Best J. R. Miller P. H. Jones L. L. (2009). Executive functions after age 5: changes and correlates. *Dev. Rev.* 29 180–200. 10.1016/j.dr.2009.05.002 20161467 PMC2792574

[B8] Biederman J. Monuteaux M. C. Doyle A. E. Seidman L. J. Wilens T. E. Ferrero F. (2004). Impact of executive function deficits and attention-deficit/hyperactivity disorder (ADHD) on academic outcomes in children. *J. Consult. Clin. Psychol.* 72 757–766. 10.1037/0022-006X.72.5.757 15482034

[B9] Blair C. Razza R. P. (2007). Relating effortful control, executive function, and false belief understanding to emerging math and literacy ability in kindergarten. *Child Dev.* 78 647–663. 10.1111/j.1467-8624.2007.01019.x 17381795

[B10] Brosschot J. F. Gerin W. Thayer J. F. (2006). The perseverative cognition hypothesis: a review of worry, prolonged stress-related physiological activation, and health. *J. Psychosom. Res.* 60 113–124. 10.1016/j.jpsychores.2005.06.074 16439263

[B11] Bull R. Espy K. A. Wiebe S. A. (2008). Short-term memory, working memory, and executive functioning in preschoolers: longitudinal predictors of mathematical achievement at age 7 years. *Dev. Neuropsychol.* 33 205–228. 10.1080/87565640801982312 18473197 PMC2729141

[B12] Byrne D. G. Davenport S. C. Mazanov J. (2007). Profiles of adolescent stress: the development of the adolescent stress questionnaire (asq). *J. Adolesc.* 30 393–416. 10.1016/j.adolescence.2006.04.004 16750846

[B13] Catalano D. Chan F. Wilson L. Chiu C. Y. Muller V. R. (2011). The buffering effect of resilience on depression among individuals with spinal cord injury: a structural equation model. *Rehabil. Psychol.* 56 200–211. 10.1037/a0024571 21843016

[B14] Chen X. Li D. Xu X. Liu J. Fu R. Cui L. (2019). School adjustment of children from rural migrant families in urban China. *J. Sch. Psychol.* 72 14–28. 10.1016/j.jsp.2018.12.003 30819459

[B15] Clark C. Prior M. Kinsella G. (2002). The relationship between executive function abilities, adaptive behaviour, and academic achievement in children with externalising behaviour problems. *J. Child Psychol. Psychiatry* 43 785–796. 10.1111/1469-7610.00084 12236613

[B16] Clark C. A. Pritchard V. E. Woodward L. J. (2010). Preschool executive functioning abilities predict early mathematics achievement. *Dev. Psychol.* 46 1176–1191. 10.1037/a0019672 20822231

[B17] Cole N. N. Nonterah C. W. Utsey S. O. Hook J. N. Hubbard R. R. Opare-Henaku A. (2015). Predictor and moderator effects of ego resilience and mindfulness on the relationship between academic stress and psychological well-being in a sample of Ghanaian college students. *J. Black Psychol.* 41 340–357. 10.1177/0095798414537939

[B18] Compas B. E. (1987). Stress and life events during childhood and adolescence. *Clin. Psychol. Rev.* 7 275–302. 10.1016/0272-7358(87)90037-7

[B19] Compas B. E. Orosan P. G. Grant K. E. (1993). Adolescent stress and coping: implications for psychopathology during adolescence. *J. Adolesc.* 16 331–349. 10.1006/jado.1993.1028 8282901

[B20] Curtis J. R. Treece P. D. Nielsen E. L. Gold J. Ciechanowski P. S. Shannon S. E. (2016). Randomized trial of communication facilitators to reduce family distress and intensity of end-of-life care. *Am. J. Respir. Criti. Care Med.* 193 154–162. 10.1164/rccm.201505-0900OC 26378963 PMC4731711

[B21] Davidovich S. Collishaw S. Thapar A. K. Harold G. Thapar A. Rice F. (2016). Do better executive functions buffer the effect of current parental depression on adolescent depressive symptoms? *J. Affect. Disord.* 199 54–64. 10.1016/j.jad.2016.03.049 27085164 PMC4871808

[B22] Diamond A. (2013). Executive functions. *Ann. Rev. Psychol.* 64 135–168. 10.1146/annurev-psych-113011-143750 23020641 PMC4084861

[B23] Duckworth A. L. Kim B. Tsukayama E. (2012). Life stress impairs self-control in early adolescence. *Front. Psychol.* 3:608. 10.3389/fpsyg.2012.00608 23443890 PMC3581033

[B24] Dupéré V. Dion E. Leventhal T. Archambault I. Crosnoe R. Janosz M. (2018). High school dropout in proximal context: the triggering role of stressful life events. *Child Dev.* 89 e107–e122. 10.1111/cdev.12792 28369807 PMC10624510

[B25] Ellis L. K. Rothbart M. K. Posner M. I. (2004). Individual differences in executive attention predict self-regulation and adolescent psychosocial behaviors. *Ann. N. Y. Acad. Sci.* 1021 337–340. 10.1196/annals.1308.041 15251906

[B26] Espy K. A. Mcdiarmid M. M. Cwik M. F. Stalets M. M. Hamby A. Senn T. E. (2004). The contribution of executive functions to emergent mathematic skills in preschool children. *Dev. Neuropsychol.* 26 465–486. 10.1207/s15326942dn2601_6 15276905

[B27] Fergus S. Zimmerman M. A. (2004). Adolescent resilience: a framework for understanding healthy development in the face of risk. *Ann. Rev. Public Health* 26 399–419. 10.1146/annurev.publhealth.26.021304.144357 15760295

[B28] Fitzpatrick C. McKinnon R. D. Blair C. B. Willoughby M. T. (2014). Do preschool executive function skills explain the school readiness gap between advantaged and disadvantaged children? *Learn. Instr.* 30 25–31. 10.1016/j.learninstruc.2013.11.003

[B29] García-Izquierdo M. Ríos-Risquez M. I. Carrillo-García C. Sabuco-Tebar E. D. L. Á (2018). The moderating role of resilience in the relationship between academic burnout and the perception of psychological health in nursing students. *Educ. Psychol.* 38 1068–1079. 10.1080/01443410.2017.1383073

[B30] Gioia G. A. Isquith P. K. Guy S. C. Kenworthy L. (2000). Test review behavior rating inventory of executive function. *Child Neuropsychol.* 6 235–238. 10.1076/chin.6.3.235.3152 11419452

[B31] Graham J. W. (2009). Missing data analysis: making it work in the real world. *Ann. Rev. Psychol.* 60 549–576. 10.1146/annurev.psych.58.110405.085530 18652544

[B32] Greenaway R. Howlin P. (2010). Dysfunctional attitudes and perfectionism and their relationship to anxious and depressive symptoms in boys with autism spectrum disorders. *J. Autism Dev. Disord.* 40 1179–1187. 10.1007/s10803-010-0977-z 20182783

[B33] Greenberg M. T. (2006). Promoting resilience in children and youth. *Ann. N. Y. Acad. Sci.* 1094 139–150. 10.1196/annals.1376.013 17347347

[B34] Han L. Zhao S. Y. Pan X. Y. Liao C. J. (2018). The impact of students with left-behind experiences on childhood: the relationship between negative life events and depression among college students in China. *Int. J. Soc. Psychiatry* 64 56–62. 10.1177/0020764017739332 29145763

[B35] Hu L. T. Bentler P. M. (1999). Cutoff criteria for fit indexes in covariance structure analysis: conventional criteria versus new alternatives. *Struct. Equ. Modeling* 6 1–55. 10.1080/10705519909540118

[B36] Huang C. Tang Y. Wang L. Xie D. Fan C. Gao W. (2014). Development of adolescent executive function scale. *Chin. J. Behav. Med. Brain Sci.* 23 463–465.

[B37] Hughes C. Ensor R. (2011). Individual differences in growth in executive function across the transition to school predict externalizing and internalizing behaviors and self-perceived academic success at 6 years of age. *J. Exp. Child Psychol.* 108 663–676. 10.1016/j.jecp.2010.06.005 20673580

[B38] Humphreys K. L. Watts E. L. Dennis E. L. King L. S. Thompson P. M. Gotlib I. H. (2018). Stressful life events, ADHD symptoms, and brain structure in early adolescence. *J. Abnorm. Child Psychol.* 10.1007/s10802-018-0443-5 [Epub ahead of print]. 29785533 PMC6249129

[B39] Jacobson L. A. Williford A. P. Pianta R. C. (2011). The role of executive function in children’s competent adjustment to middle school. *Child Neuropsychol.* 17 255–280. 10.1080/09297049.2010.535654 21246422 PMC4075458

[B40] Kim I. Kim W. S. Bae S. C. (2018). Effects of adolescents’ academic stress on school life adjustment: focusing on mediator effect of resilience. *Medico Legal Update* 18 290–295. 10.5958/0974-1283.2018.00060.9

[B41] Kramer M. S. Lydon J. Goulet L. Kahn S. R. Mcnamara H. Genest J. (2009). Stress pathways to spontaneous preterm birth: the role of stressors, psychological distress, and stress hormones. *Am. J. Epidemiol.* 169 1319–1326. 10.1093/aje/kwp061 19363098

[B42] Lafavor T. (2018). Predictors of academic success in 9-to 11-year-old homeless children: the role of executive function, social competence, and emotional control. *J. Early Adolesc.* 38 1236–1264. 10.1177/0272431616678989

[B43] Lan X. Scrimin S. Moscardino U. (2019). Perceived parental guan and school adjustment among chinese early adolescents: the moderating role of interdependent self-construal. *J. Adolesc.* 71 18–27. 10.1016/j.adolescence.2018.12.003 30586663

[B44] Lee S. Hwang S. Song Y. Lee H. (2014). Mediating effects of inter-organizational relation on the relation between resilience and school adjustment in elementary school students. *J. Fish. Mar. Sci. Educ.* 26 1217–1230.

[B45] Li P. Zhang J. Li M. Li P. Zhang Y. Xin Z. (2012). Negative life events and mental health of chinese medical students: the effect of resilience, personality and social support. *Psychiatry Res.* 196 138–141. 10.1016/j.psychres.2011.12.006 22405636

[B46] Luthar S. S. Cicchetti D. Becker B. (2000). The construct of resilience: a critical evaluation and guidelines for future work. *Child Dev.* 71 543–562. 10.1111/1467-8624.00164 10953923 PMC1885202

[B47] Maclean M. J. Taylor C. L. O’Donnell M. (2016). Pre-existing adversity, level of child protection involvement, and school attendance predict educational outcomes in a longitudinal study. *Child Abuse Negl.* 51 120–131. 10.1016/j.chiabu.2015.10.026 26626345

[B48] Masten A. S. (2014). Global perspectives on resilience in children and youth. *Child Dev.* 85 6–20. 10.1111/cdev.12205 24341286

[B49] Masten A. S. (2015). Pathways to integrated resilience science. *Psychol. Inq.* 26 187–196. 10.1080/1047840X.2015.1012041

[B50] Masten A. S. Tellegen A. (2012). Resilience in developmental psychopathology: contributions of the project competence longitudinal study. *Dev. Psychopathol.* 24 345–361. 10.1017/S095457941200003X 22559118

[B51] McAuley E. Mullen S. P. Szabo A. N. White S. M. Wójcicki T. R. Mailey E. L. (2011). Self-regulatory processes and exercise adherence in older adults: executive function and self-efficacy effects. *Am. J. Preven. Med.* 41 284–290. 10.1016/j.amepre.2011.04.014 21855742 PMC3160622

[B52] McKee J. (2017). *Executive Functions and Resilience in First-Year Undergraduate Students.* Doctoral dissertation, University of Calgary, Calgary.

[B53] Migerode F. Maes B. Buysse A. Brondeel R. (2012). Quality of life in adolescents with a disability and their parents: the mediating role of social support and resilience. *J. Dev. Phys. Disabil.* 24 487–503. 10.1007/s10882-012-9285-1

[B54] Muthén L. K. Muthén B. O. (1998/2012). *Mplus User’s Guide* 7th Edn. Los Angeles, CA: Muthén & Muthén.

[B55] Navalta C. P. Polcari A. Webster D. M. Boghossian A. Teicher M. H. (2006). Effects of childhood sexual abuse on neuropsychological and cognitive function in college women. *J. Neuropsychiatry Clin. Neurosci.* 18 45–53. 10.1176/jnp.18.1.45 16525070

[B56] Nesbitt K. T. Baker-Ward L. Willoughby M. T. (2013). Executive function mediates socio-economic and racial differences in early academic achievement. *Early Childhood Res. Q.* 28 774–783. 10.1016/j.ecresq.2013.07.005

[B57] Neuenschwander R. Röthlisberger M. Cimeli P. Roebers C. M. (2012). How do different aspects of self-regulation predict successful adaptation to school? *J. Exp. Child Psychol.* 113 353–371. 10.1016/j.jecp.2012.07.004 22920433

[B58] Ni C. Chow M. C. Jiang X. Li S. Pang S. M. (2015). Factors associated with resilience of adult survivors five years after the 2008 Sichuan earthquake in China. *PLoS One* 10:e0121033. 10.1371/journal.pone.0121033 25811775 PMC4374963

[B59] Nishikawa S. Fujisawa T. X. Kojima M. Tomoda A. (2018). Type and timing of negative life events are associated with adolescent Depression. *Front. Psychiatry* 9:41. 10.3389/fpsyt.2018.00041 29491843 PMC5817059

[B60] Piccolo L. R. Noble K. G. (2017). Perceived stress is associated with smaller hippocampal volume in adolescence. *Psychophysiology* 55:e13025. 10.1111/psyp.13025 29053191 PMC5899620

[B61] Preacher K. J. Hayes A. F. (2008). Asymptotic and resampling strategies for assessing and comparing indirect effects in multiple mediator models. *Behav. Res. Methods* 40 879–891. 10.3758/BRM.40.3.879 18697684

[B62] Pukay-Martin N. D. Cristiani S. A. Saveanu R. Bornstein R. A. (2003). The relationship between stressful life events and cognitive function in HIV-infected men. *J. Neuropsychiatry* 15 436–441. 10.1176/jnp.15.4.436 14627770

[B63] Reising M. M. Bettis A. H. Dunbar J. P. Watson K. H. Gruhn M. Hoskinson K. R. (2018). Stress, coping, executive function, and brain activation in adolescent offspring of depressed and nondepressed mothers. *Child Neuropsychol.* 24 638–656. 10.1080/09297049.2017.1307950 28349772 PMC6529941

[B64] Rike P. O. Johansen H. J. Ulleberg P. Lundqvist A. Schanke A. K. (2015). Exploring associations between self-reported executive functions, impulsive personality traits, driving self-efficacy, and functional abilities in driver behaviour after brain injury. *Transp. Res. Part F Traffic Psychol. Behav.* 29 34–47. 10.1016/j.trf.2015.01.004

[B65] Rod N. H. Grønbaek M. Schnohr P. Prescott E. Kristensen T. S. (2009). Perceived stress as a risk factor for changes in health behaviour and cardiac risk profile: a longitudinal study. *J. Intern. Med.* 266 467–475. 10.1111/j.1365-2796.2009.02124.x 19570055

[B66] Saleh A. Potter G. G. McQuoid D. R. Boyd B. Turner R. MacFall J. R. (2017). Effects of early life stress on depression, cognitive performance and brain morphology. *Psychol. Med.* 47 171–181. 10.1017/S0033291716002403 27682320 PMC5195852

[B67] Sandler I. N. Tein J. Y. West S. G. (1994). Coping, stress, and the psychological symptoms of children of divorce: a cross-sectional and longitudinal study. *Child Dev.* 65 1744–1763. 10.2307/1131291 7859552

[B68] Schafer J. L. Graham J. W. (2002). Missing data: our view of the state of the art. *Psychol. Methods* 7 147–177. 10.1037/1082-989X.7.2.147 12090408

[B69] Seery M. D. (2011). Resilience: a silver lining to experiencing adverse life events? *Curr. Dir. Psychol. Sci.* 20 390–394. 10.1177/0963721411424740

[B70] Sung Y. T. Huang L. Y. Tseng F. L. Chang K. E. (2014). The aspects and ability groups in which little fish perform worse than big fish: examining the big-fish-little-pond effect in the context of school tracking. *Contemp. Educ. Psychol.* 39 220–232. 10.1016/j.cedpsych.2014.05.002

[B71] Thurston I. B. Hardin R. Kamody R. C. Herbozo S. Kaufman C. (2018). The moderating role of resilience on the relationship between perceived stress and binge eating symptoms among young adult women. *Eat. Behav.* 29 114–119. 10.1016/j.eatbeh.2018.03.009 29653301

[B72] Troy A. S. Mauss I. B. (2011). *Resilience in the Face of Stress: Emotion Regulation as a Protective Factor.* Cambridge: Cambridge University Press 30–44.

[B73] Wagnild G. M. Young H. M. (1993). Development and psychometric evaluation of the resilience scale. *J. Nurs. Measur.* 1 165–178. 7850498

[B74] Welsh J. A. Nix R. L. Blair C. Bierman K. L. Nelson K. E. (2010). The development of cognitive skills and gains in academic school readiness for children from low-income families. *J. Educ. Psychol.* 102 43–53. 10.1037/a0016738 20411025 PMC2856933

[B75] Xin X. Ming Q. Zhang J. Wang Y. Liu M. Yao S. (2016). Four distinct subgroups of self-injurious behavior among Chinese adolescents: findings from a latent class analysis. *PLoS One* 11:e0158609. 10.1371/journal.pone.0158609 27392132 PMC4938421

[B76] Xin X. H. Yao S. Q. (2015). Validity and reliability of the adolescent self-rating life events checklist in middle school students. *Chin. Ment. Health J.* 29 355–360.

[B77] Yang Y. Chen Y. Zhou H. Wang L. (2012). The influence of daily stressful life events on teenage students’ social adjustment: social problem-solving ability as a mediator. *J. Psychol. Sci.* 35 1376–1382. 10.16719/j.cnki.1671-6981.2012.06.004

[B78] Zhang X. Chen X. Ran G. Ma Y. (2016). Adult children’s support and self-esteem as mediators in the relationship between attachment and subjective well-being in older adults. *Personal. Individ. Dif.* 97 229–233. 10.1016/j.paid.2016.03.062

[B79] Zheng Y.-H. Fan F. Yu C.-F. Luo T.-C. (2011). Relationship between gratitude and symptoms of post-traumatic stress disorder among adolescents: mediation of social support and resilience. *Psychol. Dev. Educ.* 27 522–528. 10.16187/j.cnki.issn1001-4918.2011.05.003

[B80] Zhu H. Lu G. Liu H. (2012). Explore the correlations among resilience, stress and school adjustment of middle school students. *Med. Soc.* 25 71–73. 10.3870/YXYSH.2012.05.025

